# Positioning pharmacists’ roles in primary health care: a discourse analysis of the compensation plan in Alberta, Canada

**DOI:** 10.1186/s12913-017-2734-x

**Published:** 2017-11-23

**Authors:** Christine A. Hughes, Rene R. Breault, Deborah Hicks, Theresa J. Schindel

**Affiliations:** 1 Faculty of Pharmacy and Pharmaceutical Sciences, University of Alberta, 3-171 Edmonton Clinic Health Academy, 11405 87 Avenue NW, Edmonton, AB T6G 1C9 Canada; 2 School of Library, Archival and Information Studies, The University of British Columbia, 470 1961 East Mall, Vancouver, BC V6T 1Z1 Canada

**Keywords:** Compensation, Pharmacy services, Community pharmacy, Pharmacist roles, Primary health care, Discourse analysis, Social positioning theory

## Abstract

**Background:**

A comprehensive Compensation Plan for pharmacy services delivered by community pharmacists was implemented in Alberta, Canada in July 2012. Services covered by the Compensation Plan include care planning services, prescribing services such as adapting prescriptions, and administering a drug or publicly-funded vaccine by injection. Understanding how the Compensation Plan was framed and communicated provides insight into the roles of pharmacists and the potential influence of language on the implementation of services covered by the Compensation Plan by Albertan pharmacists. The objective of this study is to examine the positioning of pharmacists’ roles in documents used to communicate the Compensation Plan to Albertan pharmacists and other audiences.

**Methods:**

Publicly available documents related to the Compensation Plan, such as news releases or reports, published between January 2012 and December 2015 were obtained from websites such as the Government of Alberta, Alberta Blue Cross, the Alberta College of Pharmacists, the Alberta Pharmacists’ Association, and the Blueprint for Pharmacy. Searches of the Canadian Newsstand database and Google identified additional documents. Discourse analysis was performed using social positioning theory to explore how pharmacists’ roles were constructed in communications about the Compensation Plan.

**Results:**

In total, 65 publicly available documents were included in the analysis. The Compensation Plan was put forward as a framework for payment for professional services and formal legitimization of pharmacists’ changing professional roles. The discourse associated with the Compensation Plan positioned pharmacists’ roles as: (1) expanding to include services such as medication management for chronic diseases, (2) contributing to primary health care by providing access to services such as prescription renewals and immunizations, and (3) collaborating with other health care team members. Pharmacists’ changing roles were positioned in alignment with the aims of primary health care.

**Conclusions:**

Social positioning theory provides a useful lens to examine the dynamic and evolving roles of pharmacists. This study provides insight into how communications regarding the Compensation Plan in Alberta, Canada positioned pharmacists’ changing roles in the broader context of changes to primary health care delivery. Our findings may be useful for other jurisdictions considering implementation of remunerated clinical services provided by pharmacists.

**Electronic supplementary material:**

The online version of this article (10.1186/s12913-017-2734-x) contains supplementary material, which is available to authorized users.

## Background

In many parts of the world, pharmacy practice is undergoing a paradigm shift that highlights new roles and responsibilities in the provision of patient care. Pharmacists, as the first point of contact for the public, are becoming increasingly involved in primary health care, providing access to medications and chronic disease management services [1, 2]. They are also involved in public health through delivery of smoking cessation, cardiovascular screening, and immunization programs [3]. Most studies examining the shift to a patient care-focused practice indicate a willingness amongst community pharmacists to provide more clinical services [4, 5]. In a study of pharmacists’ intentions to provide new medication therapy management services reimbursed under Medicare in the United States, the results were, overall, positive, and most agreed they had sufficient training [6]. However, lack of remuneration is often recognized as a significant barrier to expansion of new services provided by pharmacists [7–9]. In addition to poor collaboration with other health care professionals and a shortage of shared vision about the various services provided by pharmacists, lack of remuneration has been identified as a major contributor to the underutilization of pharmacists’ services [10]. Remuneration, among other strategies, may advance the profession and enhance the role of community pharmacists as primary health care providers [9, 11].

In Canada, momentum for primary health care reform was established in the late 1990’s, precipitated in part due to growing concern about access to and quality of health care [12] as well as findings from reports on the publicly funded Canadian health care system [13]. Membership of pharmacists on health care teams, and collaboration between health care professionals, was deemed important for primary health care reform [13]. In order to align pharmacy practice with the health care needs of Canadian patients, the Task Force on a Blueprint for Pharmacy developed a vision for pharmacy practice in Canada [14]. As part of the vision for pharmacy, pharmacists would practice to the full extent of their knowledge and skills and manage drug therapy in collaboration with patients and other health care providers [14]. The Blueprint for Pharmacy also stated pharmacists’ services should be compensated based on expertise and complexity of care [14]. Over the past decade, legislative changes have facilitated expansion of the scope of pharmacy practice in Canada, although approaches have differed by province or territory [15, 16].

In the province of Alberta, with its population of approximately four million, incremental changes have occurred over time to support increased involvement of pharmacists in the delivery of patient-centred services. Pharmacists were permitted access to provincial electronic health records in 2006, including laboratory tests and records of dispensed medications [17]. This was followed in 2007 by enactment of regulations that allow pharmacists to prescribe and administer drugs by injection [18]. A framework for pharmacist prescribing that includes initial access prescribing was developed by the Alberta College of Pharmacists, the regulatory organization responsible for the quality of pharmacy practice [19]. In July 2012, a government-funded Compensation Plan for Pharmacy Services provided by community pharmacists (Compensation Plan hereafter) was introduced following negotiations with the Alberta Pharmacists’ Association, the advocacy organization for pharmacists. The Plan is complementary to the scope of practice in Alberta and is commonly referred to by pharmacists as the Pharmacy Services Framework [20, 21]. Prior to introduction of the Compensation Plan, pharmacies were remunerated based on government negotiated dispensing fees. While pharmacies continue to receive dispensing fees, expanded services covered by the Compensation Plan include: care planning services and patient follow-up; prescribing services that require patient assessment such as trial prescriptions, refusal to fill a prescription, adapting or renewing a prescription, and independent prescribing to initiate or manage ongoing therapy; and administering drugs or publicly-funded vaccines by injection. The services covered by the Compensation Plan were revised in 2014 to include payment for additional services. A full description of the development of the Compensation Plan, services covered as well as associated fees, has been previously published [22]. The Compensation Plan is one of the most comprehensive fee schedules available for remuneration of clinical pharmacy services in Canada [15]. Alberta Blue Cross administers payment for pharmacy services on behalf of the government.

In addition to Canada, remuneration models to pay for pharmacy services have been implemented in other parts of the world including the United States, United Kingdom (UK), Australia, and New Zealand [23]. A systematic review by Houle et al. found that the most common remunerated service was medication reviews with or without development of a care plan, and that evaluation data for remunerated services was limited [23]. The UK was one of the first countries to expand the scope of pharmacist practice, and in 2005 introduced a new contractual framework for community pharmacy [24]. Within this framework, there are three different types of services: essential (e.g. dispensing, disposal of unwanted medicines), enhanced (e.g. minor ailment prescribing, smoking cessation programs) and advanced (e.g. medication use review). Research has shown that in many cases these pharmacy services are effective [25]. Services such as medication use reviews are generally positively viewed by patients and pharmacists [26, 27]; however research has shown less positive views by general practitioners, in part due to perceptions regarding duplication of work [28]. Observational studies that have explored patient-pharmacist interactions as part of medication use review consultations have noted challenges integrating these services into routine workload, and lack of patient awareness about the consultation [29].

Given legislative changes to enable expanded scope of pharmacist practice and introduction of a Compensation Plan in Alberta, research is needed to evaluate how pharmacists are providing patient care services as well as the perceived value of these services to patients, pharmacists, and other healthcare providers. The current study is part of a larger project looking at how pharmacists are providing care planning services in Alberta. Documents such as government communications and trade and newspaper articles are important sources of data when examining the context and implementation of government policy. Governments write policy with a certain target audience in mind; however, this information is also available to other, non-target audiences [30, 31]. For instance, documents written about the Compensation Plan provide pharmacists with important information about the clinical pharmacy services for which they can be compensated; at the same time, it informs pharmacists and the general public about expectations for and changes in pharmacists’ professional roles.

Language constructs the social world because it influences perceptions and actions [32]. Various text-based analytical approaches have been used to examine how government policies affect patient care, the effect of the media on how government policies are understood by the general public, and how pharmacists’ professional roles are conceptualized by both pharmacists and the general public [33–40]. The objective of this study is to examine the positioning of pharmacists’ roles in documents used in the communication of the Compensation Plan to Albertan pharmacists and other audiences. Understanding how the Compensation Plan was framed and communicated provides insight into pharmacists’ roles and helps to identify factors that influence implementation of the Compensation Plan by Albertan pharmacists.

## Methods

### Data sources

Publicly available documents were collected from the websites of the Government of Alberta, Alberta Blue Cross, the Alberta College of Pharmacists, the Alberta Pharmacists’ Association, and the Blueprint for Pharmacy web site. Each web site was searched for the phrases “Pharmacy Services Framework” and “Compensation Plan for Pharmacy Services”, and for the search string pharm* AND compensat* AND reimburse* AND Alberta. In addition, Canadian Newsstand, a database of major Canadian newspapers, was searched using the same phrases and search string. The search terms were based on a literature review of compensation for pharmacy services. The term ‘pharmacy services framework’ was included because of its common use by pharmacists to describe the Compensation Plan. Documents were included in the data set if they were published between 2012 and 2015. The time frame established for the data set was based on the publication date of the first document describing the Compensation Plan (2012) and concluded just prior to conducting the search (December 31, 2015). Newspaper articles included articles from the two major Albertan daily papers (the *Edmonton Journal* and *Calgary Herald*), a variety of smaller weekly publications aimed at rural communities, and news wire feed stories. Lastly, a Google search for the same phrases and search string was performed and relevant documents included in the final data set. Because Google customizes search results based on location and search history, this search was performed on two different computers, one on the University of Alberta’s campus and one off campus, on the same day (January 6, 2016). Searches were conducted over a condensed period, January 4 to 6, 2016.

### Theoretical framework

The theoretical framework informing this study is based on social positioning theory. Social positioning theory is associated with social constructionism, which recognizes the central role of language in shaping individuals’ understanding, their actions, and what they are able to accomplish in society [41]. Davies and Harré introduced social positioning as a way to explore individuals’ societal roles through language or discourse [42]. Harré and van Langenhove define the core of social positioning theory as a positioning triangle. The positioning triangle represents “a dynamic stability between actors’ positions, the social force of what they say and do, and the storylines that are instantiated in the sayings and doings of each episode” [43] p. 10. Social positioning theory acknowledges societal roles, such as the professional roles of pharmacists, as dynamic and fluid in relation to others as opposed to the more traditional, static concept of roles [42]. This theory also recognizes the possibility of multiple roles and allows exploration of roles in changing relationships within a specific context [43]. This approach aligns well with research exploring shifts in pharmacists’ roles and responsibilities in the provision of patient care. While social positioning theory originated in the field of social psychology as a tool for researching interpersonal relationships, more recently researchers have applied it in studies of health professionals’ identity [40, 44], midwifery [45], and psychiatry [46].

Positioning is the process of assigning attributes to an individual or a group. It refers to the way language is used to enable certain actions through assignment of roles, duties, or obligations. Positions affect the repertoire of actions, or roles, to which an individual has access. At the same time, positioning may also restrict activities or roles or establish what actions are possible in society. Positioning is enacted through different types of positioning including self-positioning, self-repositioning, and other positioning [43]. These acts of positioning may be intentional or unintentional. Self-positioning is usually unintentional in that it is not a conscious act. For example, an individual may position oneself as a health care professional by wearing a white coat. Self-repositioning and positioning others are always intentional. An example of intentional positioning is when an individual states that someone has credentials, positioning that individual as “qualified” for a role. In this study, the concept of positioning refers to how pharmacists are positioned, or made to fit role expectations, by themselves, their colleagues, their patients, media, or others in society. Positioning happens through spoken and written language such as conversations, professional documents, and through media representations in confronting social expectations [41]. The concept of positioning offers a framework to explore how communication about the Compensation Plan constructs pharmacists’ roles.

### Analysis

The discourse analysis method is associated with social constructionist research and positioning theory because of its focus on spoken and written language. This method, based on Potter and Wetherell [32], was used to analyze the intentional positioning of pharmacists’ roles from various perspectives, including those of policy makers, professional organizations, pharmacists, and public audiences. The discourse analysis approach used in this study examines the range of discourses that construct the role of pharmacists within the context of the Compensation Plan. The analysis consisted of two phases. The entire data set was first analyzed by one research team member to identify relevant passages and discourse categories. During the analysis, attention was paid to how pharmacists’ roles and the Compensation Plan were presented, or positioned by others, and how pharmacists wrote about their roles and the Plan, or self-positioned and self-repositioned, in the publications of their professional and regulatory organizations, government, and media reports. The data were re-read to identify variations and consistencies in the discourse related to the Compensation Plan [32]. Initially, eight discourse categories were identified. Following this initial analysis, the second phase of analysis engaged the entire research team to review and discuss the data set and the eight categories. Concept of the positioning triangle were applied to identify positions (expectations or roles of pharmacists), acts (the social force of what said or done), and storylines (narratives representing meaning of what was said or done) [43]. The second phase of analysis resulted in three positioning themes. NVivo version 10 was used to code and store data.

Differences in interpretation were reconciled through team discussion and analysis continued until consensus was reached. A limitation associated with this method is that the analysis is based on the language contained in the data and not on the intent of the speaker. A common concern of discourse analysis approaches relates to trustworthiness of research process to ensure results represent a rigorous interpretation of the data and not the opinions of the researchers [47]. Reflexivity was addressed through disclosing personal views and positions on the Compensation Plan (i.e. supportive of compensation for patient care services), pharmacists’ roles (i.e. active in research on changing roles), considering the influence of professions, government, and media discourse on views of pharmacists, public and other audiences, and acknowledging the limitations associated with the availability of documents and the depth of coverage of a new Compensation Plan. Three members of the research team are associated with the pharmacy profession. Two research team members have experience using discourse analysis methods and positioning theory. To ensure the data were analyzed in a reflexive manner, the discourse categories were initially identified by the non-pharmacist team member with expertise in library and information studies, and professional roles and identity.

## Results

### Data sources

In total, 65 publicly available documents were included in the analysis (Table 1).

**Table 1:** Summary of data sources

Source	Number of documents	Primary audience
Alberta Blue Cross	14	Pharmacists
Alberta College of Pharmacists	5	Pharmacists
Alberta Government	11	Other
Alberta Pharmacists’ Association	2	Other
Canadian Pharmacists Association/ Blueprint for Pharmacy	9	Pharmacists
Journal articles	2	Pharmacists
News media	20	Other
Other websites	2	Pharmacists

Approximately half of the documents were written primarily for an audience of professional pharmacists (32 documents). The remainder consisted of newspaper articles (20 documents), and documents, such as the report from the Auditor General of Alberta, written for other audiences, including the general public, other health care professionals and patients (13 documents) (see Additional file 1).

### Positioning themes

Three positioning themes emerged from the data: expanding roles of pharmacists, contributing to primary health care, and facilitating collaboration. These three inter-related themes position pharmacists’ present and potential roles as primary health care service providers within a broader storyline of primary health care. Representative quotes and sources, including actors such as government and pharmacists, are provided for each of the positioning themes (Tables 2, 3, and 4).

**Table 2:** Sample of data representing expanding roles of pharmacists

Quote	Source (Actor)	Source	Year
Together with other pharmacy stakeholders and the Alberta government, we will move forward with fully implementing Alberta’s new Pharmacy Services Framework later this year. This will ensure community pharmacists are in the best position to grow in their role as coordinators of drug therapy management that results in better patient health outcomes.	Alberta Pharmacists’ Association (Profession)	[51]	2012
The Ministry of Health, along with pharmacy representatives and the Alberta Pharmacists’ Association, has developed a Compensation Plan for pharmacy services to compensate Alberta’s pharmacists for offering these pharmacy services to Albertans, and provide them with the opportunity for more involvement in the health outcomes of their clients and patients.	Alberta Blue Cross	[53]	2012
The role of the pharmacist in Alberta is changing… We are continuing to enhance this framework and are encouraging pharmacists to increase their participation in health care teams like primary care networks or family care clinics.	News Media (Government)	[52]	2013
The services are meant to complement those offered by doctors, not compete with them.	News Media (Profession, Government)	[54]	2012
It’s an agreement that recognizes the expanding role of pharmacists and provides compensation to allow pharmacists to provide additional services far and beyond just filling prescriptions.	News Media (Government, Minister of Health)	[49]	2014
This really changes how pharmacists are practising, shifting from a product-based to a more assessment-based profession.	News Media (Profession)	[49]	2014

**Table 3:** Sample of data representing contributing to primary health care

Quote	Source (Actor)	Source	Year
“This announcement is the future of pharmacy services in Alberta,” Horne said at a news conference at the University of Alberta Hospital. “It’s an important step because it’s focused on making primary health care more convenient and more accessible for Albertans.”	News Media (Government, Minister of Health)	[56]	2012
The Compensation Plan for Pharmacy Services…aimed to improve patient access to health professionals, increase efficiencies in health care delivery, provide incentives for patient focused pharmacy care in the community, and increase the capacity of the health care system overall by better utilizing our health professionals	Alberta Government	[21]	2014
By supporting pharmacists to work the full scope it is hoped to free up doctors’ appointment time and shorten wait times for those who need to see a physician.	News Media	[59]	2012
There are over 4300 pharmacists licensed in Alberta who are readily accessible to help Albertans with their medication needs close to home. Pharmacists have a key role to play in the health of Albertans and offer a broad range of services to ensure the best health outcomes for their patients. The new framework will improve patient health care access and increase efficiencies in health care delivery.	Alberta Pharmacists’ Association (Profession)	[57]	nd
You now have more choices for where you get your health services: you can still use your family physician or you can go to your local pharmacist to have these services performed. Overall this change will mean more convenience for you, more timely access to medications and a more efficient use of a valuable health care resource.	Government (Alberta Health)	[60]	nd
Albertans will soon be able to get their prescriptions renewed at a pharmacy without having to go see a doctor first, the province announced Monday.	News Media	[56]	2012
The objective is to free up doctors and nurses more as patients may not need to see them for everything.	News Media (Pharmacist)	[61]	2014

**Table 4:** Sample of data representing facilitating collaboration

Quote	Source (Actor)	Source	Year
We want to shift the role of pharmacists from a simple dispenser of drugs to a more-integrated health-care professional.	News Media (Government, Minister of Health)	[56]	2012
In 2007, the Alberta government amended the Pharmacists Regulation of the Health Professions Act to allow pharmacists to renew, modify and in some cases prescribe medications in accordance with educational requirements and quality standards set by the Alberta College of Pharmacists. Services are provided in consultation with a patient’s physician.	Alberta Government	[51]	2012
Pharmacists will renew and modify prescriptions in consultation with physicians.	News Media	[56]	2012
Pharmacists will continue to work closely with patients and their health care teams, including physicians, so that team members are appropriately informed about decisions made by pharmacists. Inversely, it is important that pharmacists are informed by other health team members about decisions that affect the drug therapy patients require.	Alberta College of Pharmacists (Profession)	[62]	2012
Be team oriented – Intraprofessional collaboration is as important as interprofessional collaboration. Help both your pharmacy team and your colleagues in other health professions know how your practice will be changing, how you want to work together, and how these changes will benefit patients.	Alberta College of Pharmacists (Profession)	[50]	2012
Over the last few years, pharmacists have taken on a larger and more direct role in our health care system. Today pharmacists are becoming full partners in that system.	News Media (Government, Minister of Health)	[64]	2014
It sets up pharmacists to use their own discretion and their own professional opinion based on patient need. It’s all about assessing what’s good for the patient and following guidelines.	News Media (Pharmacist)	[59]	2012

#### Expanding roles of pharmacists

As its formal name suggests, the Compensation Plan for Pharmacy Services was presented to pharmacists as a plan to recognize changes in the roles of pharmacists and in how pharmacies are paid for different services. The Compensation Plan provided compensation for services for which Albertan pharmacists had had legal authorization to perform since 2007, and which were previously not compensated. By recognizing expanding professional roles through formal compensation, the pharmacists were positioned as possessing unique and valuable professional expertise and abilities to provide services such as renewing prescriptions, administering drugs by injection, developing comprehensive care plans, and managing medication therapy [48]. The changes in pharmacists’ roles were self-positioned by pharmacists as a shift from providing services based on products to providing services based on assessment of patients [49].

The documents from the Alberta College of Pharmacists positioned the Compensation Plan as a way for pharmacists to develop their practice further, which can be seen in this example from the Alberta College of Pharmacists (ACP) “Do you want to take advantage of the opportunities presented by the new pharmacy services framework, but don’t really know where to begin? Here are a few tips and ACP resources to get you started” [50]. This positioning echoed communications from the Alberta Government which reinforced the different ways the Compensation Plan could enhance pharmacists’ professional practice. For instance, the Alberta Government described the Compensation Plan as a means for pharmacists “to grow in their role as coordinators of drug therapy management that results in better patient health outcomes” [51]. Thus, following the Alberta Government, the Alberta College of Pharmacists’ documentation also positioned pharmacists’ roles and services as expanding.

In the documents written for other audiences, attention was paid to the role of the Compensation Plan in helping patients, the government, and other health care professionals recognize and understand pharmacists’ expanded scope of practice. In addition to providing payment for services already provided by pharmacists, the Compensation Plan supported pharmacists in their efforts to expand their roles beyond traditional services known to the public such as filling prescriptions [49], increase participation in health care teams [52], and become more involved in the care of patients [53]. Pharmacists’ roles related to prescribing services were positioned as complementary to services offered by physicians [54]. This positioning had the effect of highlighting pharmacists’ expanding professional roles to the general public.

#### Contributing to primary health care

Similar to the positioning described in the Expanding Roles of Pharmacist theme above, the Contributing to Primary Health Care theme also positioned pharmacists’ evolving professional roles as an important component of Albertans’ health care. The changes in scope of practice, particularly with respect to prescribing and administering drugs by injection, not just in Alberta but across Canada, “positioned pharmacists to play a larger role in primary care” [55] p.1. However, while the Expanding Roles of Pharmacist theme highlighted individual pharmacists’ professional skills and expertise, the Contributing to Primary Health Care theme focused on the role pharmacists played in the larger health care system. Along with recognition of the expanding roles and professional services of pharmacists in Alberta, the Compensation Plan represented a means to advance primary health care reform by increasing access to services, improving health outcomes, and reducing costs in the health care system. An elected member of government framed pharmacists’ changing roles as “part of a larger framework aimed at helping Albertans have more access to the health care they need” [52]. In the Ministerial Order outlining the Compensation Plan, the Government of Alberta described its desired outcomes for the Plan to “improve patient access to health professionals, increase efficiencies in health care delivery, provide incentives for patient focused pharmacy care in the community, and increase the capacity of the health care system overall by better utilizing our health professionals” [21] p. i.

In the documents accessible to public audiences, improved accessibility, patient choice, and convenience to health care professionals were highlighted as benefits of the Plan [56]. In particular, within this theme pharmacists were positioned as accessible primary health care service providers. The Minister of Health and Wellness, Mr. Horne, emphasized accessibility of pharmacists noting that announcement of the Compensation Plan represented “opening 1,000 new locations to get prescriptions renewed” [51]. Payment for services would provide incentive for pharmacists to provide services [21] and improve accessibility for patients especially those in rural and remote communities [52, 56]. In these documents, services provided by pharmacists were positioned as necessary components of the overall health care strategy of the province of Alberta.

The documents written for a public audience drew attention to selected services patients need and could access at community pharmacies including administration of drug injections, prescription renewals, care planning, and prescribing of medication in certain situations including a medical emergency [57, 58]. Media stories highlighted positive outcomes of improved patient choice and accessibility to health care, changes which may potentially translate into more efficient use of physicians’ time and expertise [59]. Information provided to the public emphasized choice, convenience, and cost savings: “You now have more choices for where you get your health services: you can still use your family physician or you can go to your local pharmacist to have these services performed. Overall this change will mean more convenience for you, more timely access to medications and a more efficient use of a valuable health care resource” [60]. Pharmacists, therefore, were positioned as not only an important part of the overall health care system, but a convenient and efficient health care option for patients.

Pharmacists similarly positioned themselves as vital to Albertans’ health care and embraced the government’s positioning them as being able to increase efficiencies in the health care system. For instance, pharmacists, represented by their provincial association, acknowledged that providing additional clinical pharmacy services could contribute to patient outcomes [57], create efficiencies by improving patients’ access to frontline health care professionals [57], and free up time for nurses and physicians to provide other patient care services [56, 61].

#### Facilitating collaboration

The profession framed the Compensation Plan as supporting pharmacists in their collaborations with patients and other healthcare professionals. For pharmacists, the collaboration described by the Compensation Plan confirmed how they had been repositioning their roles following legislative changes that had previously expanded the scope of pharmacy practice. For instance, in its introduction of the Compensation Plan to its membership, the Alberta College of Pharmacists acknowledged that the collaboration described by the Plan was already occurring, stating “pharmacists will continue to work closely with patients and their health care teams, including physicians, so that team members are appropriately informed about decisions made by pharmacists” [62]. The profession positioned pharmacists’ roles as facilitating collaboration, or sharing information, in two respects. First, to share information about services provided to patients including drug therapy decisions [62] and second to inform physicians and others about pharmacists’ expanding roles and how professionals work together to benefit patient care [50]. Pharmacists’ roles were positioned in relation to information sharing to facilitate interprofessional collaboration.

The aims of interprofessional collaboration were associated with improving the quality of primary care services and patient outcomes [63]. These aims echoed results from the Contributing to Primary Health Care theme; however, this theme added a focus on collaboration with patients to ensure their health care goals were met. For instance, in an informational webpage for the public the Alberta Pharmacists’ Association encouraged patients to work collaboratively with pharmacists for services such as creating a Comprehensive Annual Care Plan. The information emphasized that the Comprehensive Annual Care Plan and other services, including prescription renewals, were provided by the pharmacist in collaboration with the family physician [57]. Pharmacists were positioned as collaborating, or consulting, with physicians when providing services or making drug management decisions [51, 56]. The main message was that collaboration was essential for optimal patient care. By maintaining clear paths of communication among health care team members and patients, pharmacists contributed to patient care. This enabled pharmacists to self-reposition themselves as primary health care providers focused on patient needs [59]. This self-repositioning was supported by the government in its official documentation of the Compensation Plan and in its communications with the media. For instance, in 2014 Alberta’s Minister for Health and Wellness stated in a news conference that “pharmacists are becoming full partners in that system” [64]. The statement positioned pharmacists as more fully integrated in the health care system, taking on larger roles, and delivering team-based primary health care services [56, 64].

The Compensation Plan recognized expanding professional roles and greater professional autonomy of pharmacists to make health care decisions; however, by underscoring the collaborative nature of pharmacists’ expanded scope of practice pharmacists were positioned as supportive team members in achieving efficiencies in the health care system and improved outcomes for patients.

## Discussion

Our study examined documents used to communicate information about the Compensation Plan for Pharmacy Services to Albertan pharmacists and other audiences. Social positioning theory provided a lens through which to carry out our analysis; it is about “how people use words to locate themselves and others” [65]. Our findings provide insight into the dynamic and evolving roles of pharmacists. Three inter-related positioning themes highlighted pharmacists’ present and emerging roles in the context of the Compensation Plan within a storyline of primary health care. The discourse associated with the Compensation Plan positioned pharmacists’ roles as: (1) expanding to include services such as medication management for chronic diseases, (2) contributing to primary health care by providing access to services such as prescription renewals and immunizations, and (3) collaborating with other health care team members to improve patient outcomes. Pharmacists’ changing roles were positioned in alignment with the aims of primary health care, including access to services, quality and safety of care, coordination of services, prevention and management of chronic diseases, and cost of services [12].

Expansion of community pharmacists’ roles beyond dispensing and compounding of medications has gradually occurred over the past 20–30 years, starting with the introduction of pharmaceutical care in the early 1990’s [66]. The shift from a product focus to patient-care related services has occurred in Alberta through a series of incremental legislative changes starting with increased access to patient information through health records and progressing to the ability to order laboratory tests, administer drugs by injection, and prescribe in a range of situations (including renewing and adapting prescriptions, prescribing in an emergency, and prescribing on initial access) [19, 22]. The importance of incremental changes to legitimize changes in nursing practice was reported by Goodrick and Reay [67]. They found that legitimization of new roles required incremental development of an argument over time and a simultaneous effort to retain the legitimacy of former roles. By focusing on pharmacists’ educational backgrounds, professional experiences, and previously established professional frameworks, such as codes of ethics and Standards of Practice, the Compensation Plan contributed to this incremental change in pharmacists’ roles and the services they offer and reinforced the importance of their former roles as dispensers of drugs. To pharmacists, the Compensation Plan was presented as a means of supporting evolution of pharmacists’ practices whereas for the public communication focused on access to primary health care services associated with pharmacists’ expanded roles.

Community pharmacists have been viewed as one of the most accessible health care providers, but lack of clinical autonomy and remuneration based on dispensing a product have been historical challenges in utilizing pharmacists more effectively in the delivery of primary care. The Compensation Plan helped position pharmacists as providers of primary health care delivery with overall goals of improving access to services, and offering choice and convenience for patients. Communications highlighted that pharmacist services covered by the Compensation Plan complemented those of physicians. The repositioning of pharmacists’ roles in primary care is similar to what has occurred in other countries. For example, in the UK, pharmacy organizations were the main actors in promoting the reprofessionalization of pharmacy over the past couple of decades, including redefining community pharmacy’s role in the primary health care team [68]. The government in the UK was supportive of expansion of community pharmacists’ roles through a series of reports that highlighted the need for community pharmacists to become more involved in primary care [68–70]. Legislative changes to enable expanded scope of practice and remuneration of clinical pharmacy services have been important steps in both the UK and Canada in positioning pharmacists to take on a bigger role in the primary care arena.

Collaboration is a prominent theme in pharmacy and primary health care services research and is closely associated with safety [71–73]. The documents positioned the Compensation Plan as facilitating collaboration between pharmacists and physicians. For example, the comprehensive annual care plans compensated by the Plan were intended to be complementary to care plans developed by physicians in order to facilitate collaboration among providers. Collaborative medication reviews provided by pharmacists and primary care physicians are reported to improve prescribing and may reduce costs associated with health care services [10]. Previous research has found that collaboration is associated with improved patient care [74], new clinical services provided by prescribing pharmacists [75, 76], and legitimizing pharmacists’ prescribing [77]. However, researchers have also found that collaboration is not a routine part of practice [74]. Tannenbaum and Tsuyuki [78] called attention to the importance of physician–pharmacist communication in contexts where pharmacists provide expanded clinical services, such as prescribing. Barriers to communication due to location make conditions for collaboration between community pharmacists and physicians less than optimal [79]. Donald and colleagues [80] evaluated community pharmacists’ care of patients with cardiovascular disease. They concluded that efforts must be made to optimize communication between pharmacists and physicians and to clarify roles. Beginning in 2014, Albertan physicians were compensated for communication with pharmacists related to patient services covered by the Compensation Plan [81]. Lack of compensation is recognized as a barrier to implementing new practices for physicians, such as ordering routine tests, and to interprofessional collaboration [9, 82]. The new Compensation Plan positioned pharmacists as supporting information sharing and collaboration. To determine the effects of paying physicians, pharmacists, or other health care team members for communications in support of collaborative practice, additional research is required.

Based on the social constructionist view of language, our study demonstrated that expansion of pharmacists’ roles and scope of practice was supported by legislation and in the documents communicated by the pharmacy profession. Professional organizations, such as the Alberta Pharmacists’ Association and the Alberta College of Pharmacists, play an important part in legitimizing professional change, supporting practice innovation and facilitating acceptance of new practices [83]. With the introduction of the Compensation Plan in Alberta, pharmacists may become more actively involved in providing clinical pharmacy services and exercising their full scope of practice. Since pharmacists’ roles were positioned as expanding, contributing to primary health care, and facilitating collaboration among health care team members, pharmacists may be expected to alter their practice accordingly. Available data suggests that positioning of pharmacists’ roles through communications may have impacted changes in pharmacist practice. As an example, following introduction of the Compensation Plan in 2012, the number of Alberta pharmacists with Additional Prescribing Authorization increased dramatically from 167 to 1654 pharmacists; currently, 31% of Albertan pharmacists have this authorization [84]. Since implementation of the Compensation Plan, the most frequently provided services were those highlighted in the documents used in this study: prescription renewals, influenza immunizations, and prescription adaptations [22].

The context of this study, the unique practice environment of Albertan pharmacists, is one of its limitations. However, our findings illustrate how social positioning theory can contribute to understanding the relationship between communications and pharmacists’ professional roles. Strengths of this work include the research team’s involvement in the selection of documents and in the analysis itself, which lend credence to the trustworthiness of the results of this study [85]. The analysis involved reflexive discussions of views on compensation, interpretation of data, and co-construction of thematic categories related to pharmacists’ roles in primary health care. This work may be helpful to researchers, policy makers, and professional organizations interested in expanded roles for pharmacists or other health professionals.

## Conclusions

This study provides insight into how communications around the Compensation Plan in Alberta, Canada positioned pharmacists’ roles. Our findings draw attention to the role of compensation in recognizing the expanding roles of pharmacists, pharmacists’ contributions to primary health care, and collaborative patient care. The Compensation Plan was positioned as supporting further shifts in pharmacy practice. For the public, the Plan was framed as supporting access to primary health care services such as prescription renewals and administering injections. Using the framework of social positioning theory, we conducted an analysis of the Compensation Plan within the practice context in Alberta that provides valuable information for pharmacists. It may also inform other jurisdictions considering implementation or expansion of remunerated clinical services provided by pharmacists. The findings of this study provide a foundation for future research on how language influences practice and how clinical pharmacy services compensated by the Plan are interpreted and implemented by pharmacists.

## Additional file

Additional file 1: Data Set containing the list of documents analyzed for this study. (DOCX 30 kb)
