# Supplementary material for: Positioning pharmacists’ roles in primary health care: a discourse analysis of the compensation plan in Alberta, Canada
Source: BMC Health Serv Res. 2017 Nov 23;17:770. doi: 10.1186/s12913-017-2734-x (PMC5701384; doi:10.1186/s12913-017-2734-x)
Supplement: Additional file 1: — Data Set containing the list of documents analyzed for this study. (DOCX 30 kb) [file 12913_2017_2734_MOESM1_ESM.docx]

**Additional File: Data Set**

**Alberta Blue Cross**

1. Alberta Blue Cross. Compensation for pharmacy services. In: The Pharmacy Benefact, 346. 2012. [http://www.rxa.ca/media/24075/346_PSF.pdf . Accessed 14 Apr 2017](http://www.rxa.ca/media/24075/346_PSF.pdf%20.%20%20Accessed%2014%20Apr%202017).
2. Alberta Blue Cross. evolving a partnership to meet the needs of Albertans. In: Alberta Blue Cross Annual Report. 2012. https://www.ab.bluecross.ca/pdfs/anl_rep_2012.pdf. Accessed 14 Apr 2017.
3. Alberta Blue Cross. Alberta Health has provided additional clarity on frequency limitations for compensation of pharmacy services. In: The Pharmacy Benefact, 347. 2012. <http://www.rxa.ca/media/24078/347_Frequency_pharmacyservices.pdf>. Accessed 17 Apr 2017.
4. Alberta Blue Cross. Clarification on compensation for pharmacy services. In: The Pharmacy Benefact, 357. 2012. <http://www.rxa.ca/media/24102/357_PSF.pdf>. Accessed 17 Apr 2017.
5. Alberta Blue Cross. Pharmacy Services compensation update. In: The Pharmacy Benefact, 382. 2013. <http://www.rxa.ca/media/23919/382_CompensationUpdate.pdf>. Accessed 17 Apr 2017.
6. Alberta Blue Cross. Pharmacy services compensation update. In: The Pharmacy Benefact, 388. 2013. <http://www.rxa.ca/media/23937/388_compensationupdate.pdf>. Accessed 17 Apr 2017.
7. Alberta Blue Cross. Government of Alberta announces changes to provincial health programs. In: Connection Bulletin. 2013;1-2. <https://www.ab.bluecross.ca/pdfs/83122_connection_bulletin_spring_2013.pdf>. Accessed 14 Apr 2017.
8. Alberta Blue Cross. Signing of agreement-in-principle signals changes in prescription pricing for Albertans. In: Alberta Blue Cross Annual Report. 2013; 4. <https://www.ab.bluecross.ca/pdfs/anl_rep_2013.pdf>. Accessed 14 Apr 2017.
9. Alberta Blue Cross. Changes coming to prescription drug pricing in Alberta. In: Connection Bulletin. 2014;1-2. https://www.ab.bluecross.ca/pdfs/83219-connection-bulletin-spring2014.pdf. Accessed 14 Apr 2017.
10. Alberta Clue Cross. Alberta Blue Cross pharmaceutical services provider agreement (pharmacy). 2014. <https://www.ab.bluecross.ca/pdfs/abc_pspa.pdf>. Accessed 17 Apr 2017.
11. Alberta Blue Cross. Special announcement. In: The Pharmacy Benefact, 427. 2014. <https://www.ab.bluecross.ca/pdfs/201401_427.pdf>. Accessed 17 Apr 2017.
12. Alberta Blue Cross. Pharmacy services compensation update. In: The Pharmacy Benefact, 446. 2014. <https://www.ab.bluecross.ca/pdfs/82320.446.pdf>. Accessed 17 Apr 2017.
13. Alberta Blue Cross. Compliance verification on claims for pharmacy services. In: The Pharmacy Benefact, 493. 2014. <https://www.ab.bluecross.ca/pdfs/pharmacy-benefacts/493-compliance-verification-on-claims-for-pharmacy-services.pdf>. Accessed 17 Apr 2017.
14. Alberta Blue Cross. Reference guide for Alberta pharmacies: a resource manual for pharmacies using direct bill claims adjudication. (n.d.). <https://www.ab.bluecross.ca/pdfs/82477_pharmacy_reference_guide.pdf>. Accessed 17 Apr 2017.

**Alberta College of Pharmacists**

1. Alberta College of Pharmacists. New services framework: what can you and your patients expect? 2012. In: The Link. 2012; June: 12. <https://pharmacists.ab.ca/link-jun-12-2012>. Accessed 24 Apr 2017.
2. Alberta College of Pharmacists. Pharmacy services framework: everything old is new again. In: acpnews. 2012; July/August: 8-9. <https://pharmacists.ab.ca/sites/default/files/newsletters/ACPnewsJulyAugust-2012_0.pdf>. Accessed 18 Apr 2017.
3. Alberta College of Pharmacists. 5 tips and 5 questions to make the new pharmacy services framework work for pharmacists and patients. In: acpnews. 2012; July/August: 10-11. <https://pharmacists.ab.ca/sites/default/files/newsletters/ACPnewsJulyAugust-2012_0.pdf>. Accessed 18 Apr 2017.
4. Watson T. 2015 future of pharmacy. In: Alberta College of Pharmacists. 2015. <https://pharmacists.ab.ca/2015-future-pharmacy-tyler-watson>. Accessed 18 Apr 2017.
5. Stemplfle R. 2015 W.L. Boddy pharmacy team: Pharmacare clinical pharmacist team. In: Alberta College of Pharmacists. 2015. https://pharmacists.ab.ca/2015-wl-boddy-pharmacy-team-pharmacare-clinical-pharmacist-team. Accessed 18 Apr 2017.

**Alberta Government**

1. Alberta Health. Drug coverage and supplementary health benefits. <http://www.health.alberta.ca/services/drug-coverage-services.html>. Accessed 19 Apr 2017.
2. Alberta Health. Pharmacy fee reimbursement. <http://www.health.alberta.ca/services/pharmacy-fee-reimbursement.html>. Accessed 19 Apr 2017.
3. Alberta Health. MedRec is about keeping patients safe. <http://www.albertahealthservices.ca/info/Page9340.aspx>. Accessed 20 Apr 2017.
4. Alberta Health. Pharmacy services. <http://www.health.alberta.ca/services/pharmacy-services.html>. Accessed 19 Apr 2017.
5. Alberta Government. In: Annual report 2013-14. Health. 2013-14. <http://www.health.alberta.ca/documents/Annual-Report-14.pdf>. Accessed 19 Apr 2017.
6. Alberta Government. In: Annual report 2012-13. Health. 2012-13. <http://www.health.alberta.ca/documents/Annual-Report-13.pdf>. Accessed 19 Apr 2017.
7. Auditor General of Alberta. In: Report of the Auditor General of Alberta. Health – Chronic disease management. 2014. <http://www.oag.ab.ca/webfiles/reports/OAGSept2014Report.pdf>. Accessed 19 Apr 2017.
8. Government of Alberta. Compensation plan for pharmacy services. In: Alberta Health, Ministerial Order 23/2014. 2014. [http://www.health.alberta.ca/documents/MO-23-2014-PharmacyCompensation.pdf. Accessed 18 Apr 2017](http://www.health.alberta.ca/documents/MO-23-2014-PharmacyCompensation.pdf.%20Accessed%2018%20Apr%202017).
9. Government of Alberta. More health care options for Albertans with expanded role for pharmacists. In: announcements. 2012. http://alberta.ca/release.cfm?xID=3193577D3F312-EBAE-3E34-4AA1D8513857D09F. Accessed 19 Apr 2017.
10. Alberta Government. In: news release. 2012; Feb 13. More health care options for Albertans with expanded role for pharmacists. 2012. <https://www.alberta.ca/release.cfm?xID=3193577D3F312-EBAE-3E34-4AA1D8513857D09F>. Accessed 20 Apr 2017.
11. Alberta Government. In: news release. 2014; Feb 11. New agreement allows pharmacies to offer more services. <https://www.alberta.ca/release.cfm?xID=358405809CFC2-0EAF-8179-FDCE3985769E116C>. Accessed 20 Apr 2017.

**Alberta Pharmacists’ Association**

1. Alberta Pharmacists’ Association. For the public. (n.d.). <http://www.rxa.ca/pharmacy-services-framework/for-the-public.aspx>. Accessed 18 Apr 2017.
2. Alberta Pharmacists’ Association. Pharmacy Services Framework. In: news releases. 2012. <http://www.rxa.ca/about-rxa/news-releases/news-releases/2012/pharmacy-services-framework-and-reimbursement-finalized.aspx>. Accessed 18 Apr 2017.

**Canadian Pharmacists Association/Blueprint for Pharmacy**

1. Blueprint for Pharmacy. Comprehensive reimbursement model approved as Alberta government lowers generic prices. In: Blueprint in Motion. 2012; 3(6):2. <http://blueprintforpharmacy.ca/docs/blueprint-in-motion/blueprint-in-motion-july-2012-(2).pdf>. Accessed 16 Sept 2016.
2. Canadian Pharmacists Association. In: Blueprint for Pharmacy. 2013. Cross-jurisdiction scan of legislative and policy framework. http://blueprintforpharmacy.ca/docs/kt-tools/blueprint_policy-framework_final---january-2013.pdf. Accessed 16 Sept 2016.
3. Canadian Pharmacists Association. In: Blueprint for Pharmacy. 2013. Pharmacists’ services: Optimizing drug therapy outcomes for Canadians through patient-centred collaborative care. http://blueprintforpharmacy.ca/docs/resource-items/hciwg-pharmacy-practice-change-services_cpha_june10-2013final.pdf. Accessed 16 Sept 2016.
4. Canadian Pharmacists Association. In: Blueprint for Pharmacy. 2014. Environmental scan: Pharmacy practice legislation and policy changes across Canada. <http://blueprintforpharmacy.ca/docs/kt-tools/environmental-scan---pharmacy-practice-legislation-and-policy-changes-may-2014.pdf>. Accessed 16 Sept 2016.
5. Canadian Pharmacists Association. In: Blueprint for Pharmacy. 2014. 2014 in review: a snapshot of pharmacy news and expanded scope of practice across Canada. http://blueprintforpharmacy.ca/about/blueprint-in-motion/blueprint-in-motion---january-2015. Accessed 16 Sept 2016.
6. National Coordinating Office. Alberta’s Health Minister announces changes for pharmacy. In: Blueprint for Pharmacy. 2012. http://blueprintforpharmacy.ca/news/news-story/2012/02/16/alberta's-health-minister-announces-changes-for-pharmacy. Accessed 16 sept 2016.
7. National Coordinating Office. In: Blueprint for Pharmacy. 2012. Comprehensive reimbursement model for Alberta pharmacists. <http://blueprintforpharmacy.ca/news/news-story/2012/07/06/comprehensive-reimbursement-model-for-alberta-pharmacists>. Accessed 16 Sept 2016.
8. National Coordinating Office. In: Blueprint for Pharmacy. 2014. New pharmacy agreement announced in Alberta. http://blueprintforpharmacy.ca/resources/policy-papers-reports-and-research/2014/01/30/new-pharmacy-agreement-announced-in-alberta. Accessed 16 Sept 2016.
9. Torrance, J. A new way forward: Exploring a new pharmacist practice model. In: Blueprint in Motion. 2013; 5(1):1-19. <http://docplayer.net/8038956-Special-supplement-a-new-way-forward-exploring-a-new-pharmacist-practice-model-joshua-torrance-university-of-alberta.html>. Accessed 19 Apr 2017.

**Journal Articles**

1. Banh H. L., Cave A. So, what is holding the pharmacists back? J Pharma Health Care Sys. 2014; 1:1-2. http://www.omicsgroup.org/journals/so-what-is- holding-the-pharmacists-back-JPCHS-1-e109.pdf. Accessed 20 Apr 2017.
2. Lynas K. Reimbursement model for pharmacy service takes effect in Alberta. Can Pharm J. 2012; 145: 209. <https://www.ncbi.nlm.nih.gov/pmc/articles/PMC3567583/>. Accessed 20 Apr 2017.

**News Media**

1. Beecher J. AHS to compensate pharmacists for services. In: The Brooks Bulletin. 2012. <http://search.proquest.com.login.ezproxy.library.ualberta.ca/docview/1024620246?accountid=14474>. Accessed 20 Apr 2017.
2. Bergh R. Nothing to do with Katz. In: Calgary Herald. 2012; Nov 3. <http://search.proquest.com.login.ezproxy.library.ualberta.ca/docview/1134577975?accountid=14474>. Accessed 20 Apr 2017.
3. Brown R. Provincial pharmacy prescription welcome, say local pharmacists. In: The Brooks Bulletin. 2014; Feb 18. fromhttp://search.proquest.com.login.ezproxy.library.ualberta.ca/docview/1500144510?accountid=14474. Accessed 20 Apr 2017.
4. Dykstra M. Alberta pharmacists to offer new services starting April. In: Calgary Sun. 2014; Feb 11. <http://www.calgarysun.com/2014/02/11/alberta-pharmacists-to-offer-new-services-starting-april?token=d86f26879abbbceec9d0970718059512>. [Accessed 20 Apr 2017](http://www.edmontonsun.com/2014/02/11/alberta-pharmacists-to-offer-new-services-starting-april.%20Accessed%2020%20Apr%202017).
5. Gerein K. Pharmacists get reprieve on lower generic prices; thirty-day 'washout' will ease transition, health minister says. In: Edmonton Journal. 2013; Mar 29. <http://search.proquest.com.login.ezproxy.library.ualberta.ca/docview/1321654796?accountid=14474>. Accessed 20 Apr 2017.
6. Gerein K. Pharmacists take aim at price change; budget move on generic drugs prompts legislature protest. In: Edmonton Journal. 2013; Mar 21. <http://search.proquest.com.login.ezproxy.library.ualberta.ca/docview/1318876245?accountid=14474>. Accessed 20 Apr 2017.
7. Gerein K. A prescription for unrest. In: Edmonton Journal. 2013; May 18. <http://search.proquest.com.login.ezproxy.library.ualberta.ca/docview/1353009486/fulltext/873982D117EF48B0PQ/2?accountid=14474>. Accessed 20 Apr 2017.
8. Gerein K. Protest planned over new pharmacy rules; low generic-drug prices will hurt care, druggists say. In: Calgary Herald. 2013; Mar 21. <http://search.proquest.com.login.ezproxy.library.ualberta.ca/docview/1318878723?accountid=14474>. Accessed 20 Apr 2017.
9. Gerein K. Province, pharmacists reach dispensing-fee deal; pact also adds pay for range of new services. In: Edmonton Journal. 2014; Feb 12. <http://search.proquest.com.login.ezproxy.library.ualberta.ca/docview/1498022757?accountid=14474>. Accessed 20 Apr 2017.
10. Goudreau H. Pharmacists opening door to primary care. In: Smoky River Express. 2012; Jul 11. <http://search.proquest.com.login.ezproxy.library.ualberta.ca/docview/1024782920?accountid=14474>. Accessed 21 Apr 2017.
11. Kolafa P. Province close to deal with pharmacists. In: Inside Drumheller. 2013; Oct 11. <http://search.proquest.com.login.ezproxy.library.ualberta.ca/docview/1521042460?accountid=14474>. Accessed 21 Apr 2017.
12. Kubinec M. Concerns over generic drug prices. In: Grizzly Gazette. 2013; Apr 9. <http://search.proquest.com.login.ezproxy.library.ualberta.ca/docview/1325261223?accountid=14474>. Accessed 21 Apr 2017.
13. More health care options for Albertans with expanded role for pharmacists; up to 1,000 new locations to renew prescriptions. In: M2 Presswire. 2012; Feb 14. <http://search.proquest.com.login.ezproxy.library.ualberta.ca/docview/921280965?accountid=14474>. Accessed 21 Apr 2017.
14. O'Donnell S. Province prescribes new billing model for pharmacists. In: Calgary Herald. 2012; Jul 5.

[http://search.proquest.com.login.ezproxy.library.ualberta.ca/docview/ 1023972399?accountid=14474](http://search.proquest.com.login.ezproxy.library.ualberta.ca/docview/1023972399?accountid=14474). Accessed 21 Apr 2017.

1. Pharmacists get expanded role in system. In: Scope Newspaper. 2012; Jul 7.

<http://search.proquest.com.login.ezproxy.library.ualberta.ca/docview/1026842092?accountid=14474>. Accessed 21 Apr 2017.

1. Rieger J. Candidates questioned on major issues. In: The 40 - Mile County Commentator. 2012; Apr 3. <http://search.proquest.com.login.ezproxy.library.ualberta.ca/docview/978006287?accountid=14474>. Accessed 21 Apr 2017.
2. Rieger J. One small step for rural pharmacists, leaps and bounds left to be done. In: The 40 - Mile County Commentator. 2012; Feb 21. <http://search.proquest.com.login.ezproxy.library.ualberta.ca/docview/923154421?accountid=14474>. Accessed 21 Apr 2017.
3. Rx on the way: Alberta pharmacists able to renew prescriptions starting July 1. In: The Canadian Press. 2012; Feb 13. 13. <http://search.proquest.com.login.ezproxy.library.ualberta.ca/docview/921576726?accountid=14474>. Accessed 21 Apr 2017.
4. Schnarr J. W. New rules expanding pharmacy roles in health care*.* In: Claresholm Local Press. 2012; Sep 5. <http://search.proquest.com.login.ezproxy.library.ualberta.ca/docview/1038572238?accountid=14474>. Accessed 21 Apr 2017.
5. Vogt R. Rural pharmacies to feel effect of price reductions. In: Claresholm Local Press. 2013; Feb 13. <http://search.proquest.com.login.ezproxy.library.ualberta.ca/docview/1288145471?accountid=14474>. Accessed 21 Apr 2017.

**Other Websites**

1. Canadian Foundation for Pharmacy. Saskatchewan alumnus wins 2015 Pillar of Pharmacy award. <https://pharmacy-nutrition.usask.ca/news/2015/saskatchewan-alumnus-wins-2015-pillar-of-pharmacy-award.php>. 2015. Accessed 20 Apr 2017.
2. Wing M. Aha! moments. PharmacyU. <http://pharmacyu.ca/2014/12/21/margaret-wing-aha-moments/>. Accessed 20 Apr 2017.
